# Accumulation of microcystins in a dominant Chironomid Larvae (*Tanypus chinensis*) of a large, shallow and eutrophic Chinese lake, Lake Taihu

**DOI:** 10.1038/srep31097

**Published:** 2016-08-08

**Authors:** Qingju Xue, Xiaomei Su, Alan D. Steinman, Yongjiu Cai, Yanyan Zhao, Liqiang Xie

**Affiliations:** 1 State Key Laboratory of Lake Science and Environment, Nanjing Institute of Geography and Limnology, Chinese Academy of Sciences, 73 East Beijing Road, Nanjing 210008, China; 2 University of Chinese Academy of Sciences, Beijing, 100049, China; 3 Annis Water Resources Institute, Grand Valley State University, 740 West Shoreline Drive, Muskegon, MI 49441, USA

## Abstract

Although there have been numerous studies on microcystin (MC) accumulation in aquatic organisms recently, the bioaccumulation of MCs in relatively small sized organisms, as well as potential influencing factors, has been rarely studied. Thus, in this study, we investigated the bioaccumulation of three MC congeners (-LR, -RR and -YR) in the chironomid larvae of *Tanypus chinensis* (an excellent food source for certain fishes), the potential sources of these MCs, and potentially relevant environmental parameters over the course of one year in Lake Taihu, China. MC concentrations in *T. chinensis* varied temporally with highest concentrations during the warmest months (except August 2013) and very low concentrations during the remaining months. Among the three potential MC sources, only intracellular MCs were significantly and positively correlated with MCs in *T. chinensis*. Although MC concentrations in *T. chinensis* significantly correlated with a series of physicochemical parameters of water column, cyanobacteria species explained the most variability of MC accumulation, with the rest primarily explained by extraMC-LR. These results indicated that ingestion of MC-producing algae of cyanobacteria accounted for most of the MC that accumulated in *T. chinensis*. The high MC concentrations in *T. chinensis* may pose a potential health threat to humans through trophic transfer.

Microcystins (MCs), which are the most commonly detected cyclic heptapeptide cyanotoxin, are comprised of more than 90 congeners^1^. Of those, MC-LR, MC-RR and MC-YR are the most frequently studied congeners, and MC-LR has the strongest toxicity, followed by MC-YR and MC-RR^2^. MCs are potent hepatotoxins that can induce oxidative stress and inhibit protein phosphatases (PPs) type 1 and type 2A, leading to cell apoptosis or tumor promotion^3^,^4^. In addition, the physiochemical characteristics of MCs in certain conditions are extremely stable and persistent. For instance, MCs can remain stable for extended periods even at a temperature of 300 °C^5^, and are readily soluble in water with only small amounts being adsorbed on sediment or suspended particles^6^. Moreover, MCs are also very stable in sunlight, but can be quickly isomerized or decomposed in the presence of cyanobacterial pigment^5^,^7^. However, compared to other persistent organic pollutants (e. g., DDT), MCs are less stable and have a lower octanol-water partition coefficient (P_ow_)^8^,^9^; these characteristics suggest MCs are unlikely to be biomagnified within freshwater food webs.

Nonetheless, due to their worldwide distribution and high toxicity, MCs could accumulate in various freshwater organisms. Different aquatic organisms can accumulate substantial but variable amounts of MCs. For example, zooplankton in a coastal lagoon located in Brazil accumulated 0.3–16.4 μg/g MCs DW^10^, whereas MC concentrations in zooplankton from a eutrophic lake in the Netherlands ranged from 63 to 211 μg/g DW^11^. Chen and Xie^12^ studied MC accumulation in four bivalves from Lake Taihu (China), where whole body concentrations varied from 0.22 to 0.65 μg/g DW. In another study of two gastropods from Lake Dianchi of China, Zhang *et al.*^13^ found that a pulmonate snail (*Radix swinhoei*) accumulated higher MC concentrations than a prosobranch snail (*Margarya melanioides*). Additionally, Xie *et al.*^14^ studied MCs accumulation in fishes at different trophic levels from Lake Chao (China) and found MC content in muscle was highest in carnivorous and omnivorous fish and lowest in phytoplanktivorous fish. MC concentrations in fish varied widely (from 0.5 to 1917 μg/kg) in the study by Poste *et al.*^15^. Even different studies of MC accumulation using the same species can yield divergent results^16^,^17^. Conversely, Martins and Vasconcelos^18^ concluded that gastropods, as well as zooplankton and fish, may retain similar levels of MC. The lack of consistent findings regarding MC accumulation levels in aquatic organisms may be explained by the use of different target organisms, survival habitats and MC analysis methods.

Despite numerous studies on MC accumulation in aquatic organisms, to our knowledge only two studies have addressed chironomid larvae^19^,^20^. Chironomids are ubiquitous in most freshwater habitats, and often dominate aquatic insect communities^21^. They occupy an important position in the freshwater food web and are major food sources for certain fish and some other aquatic organisms^22^. Moreover, specific chironomid taxa can be used as bioindicators of sediment pollution^23^,^24^, and for this reason, they have been the focus of many studies examining the potential effect of pollutant concentration on aquatic life^25^,^26^,^27^. In addition, fossil assemblages of chironomid larvae have been used as indicators of climatic change^28^,^29^.

The chironomid larvae of *Tanypus chinensis* are very abundant in Lake Taihu^30^, which is a large, shallow and eutrophic lake in China. *T. chinensis*, which usually dominate in hypereutrophic conditions^31^, are not tube-builders and are freely distributed through the top few centimeters of mud. Generally, *T. chinensis* has a wide range of food sources, but detritus (including certain amounts of algae) is the most important food in adverse conditions^32^,^33^. Thus, there is the potential of MC accumulation in *T. chinensis* through predation, and the transfer of the MCs to higher trophic levels. Our study is the first report on MC accumulation in *T. chinensis*.

Our objectives in this study were to: (1) investigate the temporal variation of MC concentrations in *T. chinensis*; (2) examine the potential MC sources (sediment, intracellular from plankton, and extracellular from water column) to *T. chinensis*; and (3) explore the major environmental parameters that potentially influence MC accumulation in *T. chinensis*.

## Results

### Environmental parameters

The abiotic and biotic parameters varied over the course of the year (Table 1). Similar to other north temperate lakes, the maximum (30.93 °C) and minimum (4.61 °C) temperatures in our study area were recorded in summer and winter, respectively. pH values were much lower from November 2013 through March 2014, and again in May 2014, than in the other months. In contrast, DO followed a unimodal pattern with the maximum value recorded in March 2014. Chl-a and TSI were indicative of eutrophic conditions, and varied in tandem; SD transparency showed an inverse pattern to that of Chl-a and TSI. In addition, both TP and DTIC exhibited unimodal dynamics with the minimum and maximum values recorded in December 2013 and May 2014, respectively.

**Figure 1:**
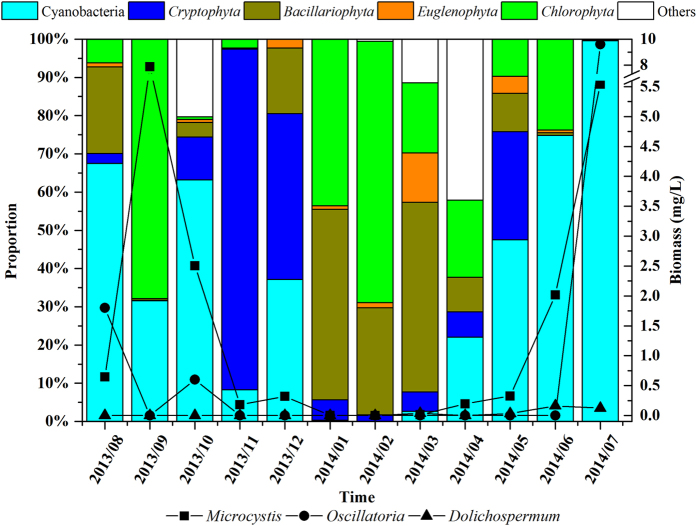
Temporal changes in taxonomic composition of phytoplankton and biomass of potential toxin–producing cyanobacteria.

One additional month (July 2014) was sampled to provide an entire year of data for the phytoplankton analysis (Fig. 1). In summer (from June to August), cyanobacteria had the greatest relative abundance at our study site, accounting for 81% of the total phytoplankton biomass with a biomass of 6.65 mg/L (mean biomass for the entire year was 2.69 mg/L). *Cryptophyta* dominated in November and also was abundant in December 2013 and May 2014. *Bacillariophyta* was observed in all months but relative abundance was greatest from January to March 2014. Although *Euglenophyta* was present in all months, its biomass was extremely low. *Chlorophyta* was abundant in September 2013 and in the winter months, with a mean annual biomass of 1.97 mg/L.

*Microcystis*, *Oscillatoria* and *Dolichospermum* were the three cyanobacteria genera identified in our samples that are capable of producing MCs (Fig. 1). Their biomass was significantly correlated with the temporal variation of CMCs (MCs in *T. chinensis*), the relative abundance of cyanobacteria was high in July 2014 and October 2013, low through most of the winter, and then began increasing again in April 2014 (Fig. 1). *Microcystis* dominated most months, except in August 2013 (*Oscillatoria*), March 2014 (*Dolichospermum*), and July 2104. In addition, *Oscillatoria* was detected only in August 2013, October 2013, and July 2014, whereas *Dolichospermum* was detected only in March and May to July 2014.

### Microcystin dynamics

**Figure 2:**
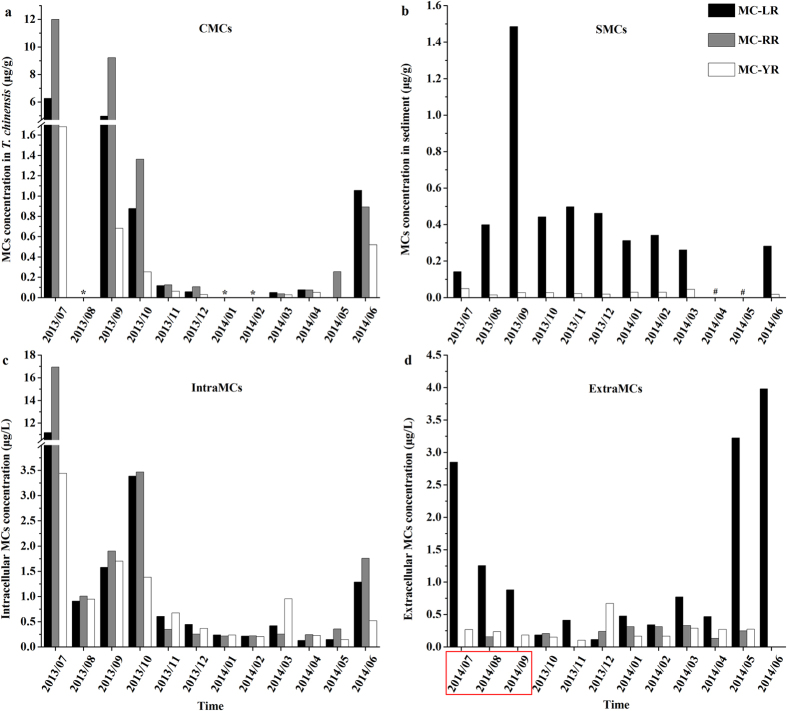
Temporal variation of MC concentrations from *T. chinensis* (**a**), sediment (**b**), intracellular cyanobacteria (**c**), and extracellular (water column; **d**). */#: undetectable/no samples. In red square: three additional months sampled in 2014; note these months are intentionally placed before the 2013 samples to keep months in consecutive order. SMCs: MCs in sediment; intraMCs: intracellular MCs; extraMCs: extracellular MCs.

CMC concentrations showed distinct temporal patterns during the study (Fig. 2a). All three MC congeners (MC-LR, MC-RR and MC-YR) reached their maximum concentrations of 6.26 μg/g, 12 μg/g and 1.68 μg/g, respectively, in July 2013. The concentrations of the three MC congeners were undetectable in the following month, but increased again in September, after which concentrations began to decrease (Fig. 2a). In July, September and October 2013, MC-RR concentrations were greatest, followed by MC-LR, and then MC-YR; they accounted for 59%, 33% and 8% of total MC, respectively. Total MC and each congener concentration decreased to very low levels between November 2013 and May 2014; none of the three congeners was detected in January or February 2014, and only MC-RR was detected in May 2014 (Fig. 2a). In June 2014, the concentrations of total MC and MC congeners began to increase, corresponding with the increase in cyanobacterial biomass (Fig. 1).

MC-LR and MC-YR were the only congeners detected in sediment (Fig. 2b). MC-LR was by far the dominant of the two (92% of total), with low MC-YR concentrations (8%) throughout the year. The concentrations of MC-LR were relatively stable throughout the year with the exception of September; sediment was not sampled in April and May 2014 (Fig. 2b).

Temporal variation of intraMCs (Fig. 2c) was similar to that of CMCs (Fig. 2a). The highest levels of intraMC were measured in July 2013, with maximum MC-LR, MC-RR and MC-YR concentrations of 11.16 μg/L, 16.94 μg/L and 3.44 μg/L, respectively. However, similar to CMCs, intraMCs declined the following month before recovering in September and October 2013. In addition, the order of most to least abundant intraMC congener concentrations was the same as that of CMCs (RR>LR>YR) between July and October (Fig. 2c). The intraMC concentrations of MC declined to low levels from November 2013 through May 2014 before starting to increase in June 2014.

In order to compensate for the missing months of extraMCs data in 2013, three additional months in 2014 were added to provide an entire year of data (Fig. 2d). The concentrations of MC-LR were low from October 2013 through April 2014, but then increased dramatically in May 2014; during May to August 2014, all MC-LR concentrations were above 1 μg/L, with a maximum value of 3.98 μg/L in June 2014; the mean value was 2.83 μg/L over these four months. MC-RR and MC-YR concentrations were much lower than MC-LR concentrations overall, with mean values of 0.16 μg/L and 0.23 μg/L, respectively.

### Correlation analysis

Results from the correlation analysis showed that all three MC congeners measured in *T. chinensis* were significantly correlated with the three intraMCs congeners, with the exception of intraMC-YR and CMC-RR (Table 2). However, no statistically significant correlations were found between total MC concentration in *T. chinensis* and sediment MC (data not shown). ExtraMC-RR was significantly and negatively correlated with all the CMC congeners and total CMC (Table 2). Among the physicochemical parameters, only pH and TSI were significantly correlated with the three CMC congeners and CTMC. DO, DTIC, and SD all were negatively correlated with CMCs, although only a few of those relationships were statistically significant (Table 2). Finally, both *Microcystis* and cyanobacteria were positively correlated with CMCs, with all *Microcystis* correlations statistically significant, whereas both *Bacillariophyta* and *Euglenophyta* were negatively correlated with CMCs.

### Stepwise multiple linear regression (MLR)

The MLR analysis included the MC congeners and TMC (total MC) accumulated in *T. chinensis* as dependent variables, and environmental parameters as independent variables (Table 3). Three parameters, cyanobacteria biomass, extraMC-LR and NO_2_^−^-N, were significantly correlated with MC-LR accumulation. Cyanobacteria biomass explained 89.2% of the variation in the dependent variable, extraMC-LR explained 10.5% of the variation, and NO_2_^−^-N explained the rest 0.3% (Table 3). In contrast to the results of CMC-LR, intraMC-RR explained the most variation (97.3%) with CMC-RR, while *Oscillatoria* biomass and TN explained only 2.4% and 0.3% of the variation, respectively, in CMC-RR. None of the algal groups was included in the stepwise regression with CMC-YR; rather, TN, NO_3_^−^-N and extraMC-YR explained 76.9%, 16.7% and 5.2% of the variation, respectively (Table 3). Cyanobacteria biomass explained the most variation (95.4%) of total MC concentrations in *T. chinensis*, followed by extraMC-LR (4.4%). VIF (variation inflation factor) values in all four models were <5 (or 3), indicating multicollinearity in these models was not an issue.

## Discussion

The larvae of the chironomid *T. chinensis* often dominate in hypereutrophic environments^31^. Despite this taxon’s abundance, and the potential for harmful algal bloom formation in hypereutrophic lakes^34^, no studies to our knowledge have addressed MC accumulation in *T. chinensis*. This phenomenon may have both ecological and human health relevance in Lake Taihu, one of the largest and most heavily used lakes in China. If *T. chinensis* is accumulating microcystins, and in turn is being consumed by other species, such as fish, which are being consumed by humans, there is a potential health risk^35^.

In the current study, there was evidence of MC accumulation by *T. chinensis*. The mean total MC concentration was 3.4 μg/g (DW), which was similar to that measured in *Limnodrilus hoffmeisteri* (3.38 μg/g DW)^36^ but lower than that measured in *Chironomus* spp. (maximum: 3.2 μg/g FW; Dry weight/Wet weight: 0.166) of Toporowska *et al.*^20^. In addition, MC accumulation in *T. chinensis* was almost several orders of magnitude higher than that accumulated in bivalvia, gastropods, shrimp and fish from the same lake^36^.

In the current study, no CMCs were detected in August 2013, despite high levels in the months before and after August. This anomaly appears to be related to the decline in intraMC concentrations in August (Fig. 2c). However, emergence of adult *T. chinensis* may also have contributed to the low August concentrations^33^. In two other studies, the highest MC levels were measured in May and June^19^,^20^, with extremely low levels in July. These differences may be caused by different emergence times by chironomid species^37^ and variable toxin-producing abilities by different cyanobacteria species.

The inverse relationship between CMC and intraMC concentrations observed in September and October 2013 may be attributable to: 1) the detoxification of early instar larvae—these instars, which dominated in biomass and abundance in September, may be more efficient at MC-YR depuration compared to MC-LR and MC-RR; 2) different amounts of toxin produced by *Microcystis* and *Oscillatoria*, either on a per capita basis or total biomass, as the biomass of *Microcystis* declined by half between September and October; and 3) the concentration of SMC-LR, another potential MC source of *T. chinensis*, increased substantially in September. Additional research is needed to assess the importance of these three possible causes.

During our survey, MC-RR was the most abundant congener in *T. chinensis* during most months, whereas MC-LR and MC-RR had similar abundances. MC-LR dominated in the other two MC sources (extraMCs and SMCs), suggesting that of the three congeners, MC-LR may be depurated the most efficiently by *T. chinensis*. A similar phenomenon was observed by Xie *et al.*^38^, who found *Sinotaia histrica* could more efficiently depurate MC-LR than MC-RR in the field. However, the opposite results also have been observed, whereby MC-LR was more resistant to degradation^16^,^39^. The lack of consistent results may be due to the use of different target species or MC sources from different habitats.

Of the three potential MC sources^20^, only intraMC concentrations were significantly and positively correlated with CMC concentrations. This is consistent with the results of another study in Lake Taihu, which focused on the variation of MC concentrations in the hepatopancreas of *Bellamya aeruginosa*^39^. Moreover, Zhang *et al.*^13^ also found similar results with *M. melanioides*, whereas the levels of MC in *R. swinhoei* were positively correlated with dissolved MCs (extraMCs). In the present study, extraMC-RR was significantly and negatively correlated with CMC concentrations, while the correlations between CMC and the other two MC congeners in the water column and SMCs were not significant. Previous studies showed that the degradation rates of MC-YR and MC-RR were several times higher than that of MC-LR^40^,^41^, which may be the primary reason for the absence of MC-RR and low concentrations of MC-YR in sediment samples.

Besides the direct influence of MC sources, abiotic factors can indirectly affect the levels of CMC through their effects on toxin-producing cyanobacteria and the metabolic activity of *T. chinensis*. High water temperature closely correlated with intraMCs in the present study, and is known to positively influence the accumulation of MC in aquatic organisms^39^,^42^. The relationships between pH and CMCs (positive) were contrary to that of DTIC. Yu *et al.*^43^ reported that the increase of DTIC and related decrease of pH could favor the dominance of nontoxic forms of *Microcystis*. Although DO was not significantly correlated with CMC concentrations in this study, it can play a decisive role in the survival^44^ and distribution of chironomids^45^.

Cyanobacteria biomass and intraMC concentrations were identified in the stepwise regression as key explanatory variables of CMC concentration, suggesting intraMCs from cyanobacterial taxa were the main source of MCs accumulated in *T. chinensis*. This was consistent with the results of correlation analysis. In addition, extraMC-LR was another important MC source of *T. chinensis*. Due to different accumulation patterns and degradation mechanisms of MCs, different species of aquatic organisms are likely to have different MC sources. For instance, MCs in *B. aeruginosa* and *M. melanioides* were primarily from intraMCs, but MCs accumulated in *R. swinhoei* were primarily influenced by extraMCs^13^,^39^. In the present study, the primary source of MCs in *T. chinensis* was intraMCs, with extraMCs contributing the remainder.

CMC-YR exhibited a different pattern in stepwise MLR analysis when compared to the other MCs (CMC-LR, -RR and CTMC); this congener was correlated with TN, NO_3_^−^-N and extraMC-YR, suggesting that its abundance was related with algal growth and possibly bacteria abundance. Thus, the two variables with the greatest explanatory power of CMC-YR abundance may also be influencing cyanobacteria/*Microcystis* growth or the abundance of MC degrading-bacteria^43^,^46^.

Finally, although MCs cannot biomagnify through the food chain in most circumstances, food chain transfer of MCs has been found in some studies^47^,^48^. CMC concentrations in the present study were extremely high during the warmer months, and given that chironomid larvae are excellent food sources for certain fishes^22^, the MCs in *T. chinensis* potentially could be transferred to higher trophic levels. Additionally, high concentrations of intraMC and extraMC also were observed in this study during the warmer months. Thus, a comprehensive water management strategy should be established as soon as possible to prevent the people and other organisms from the the potential risks of MC.

## Materials and Methods

### Study area

**Figure 3:**
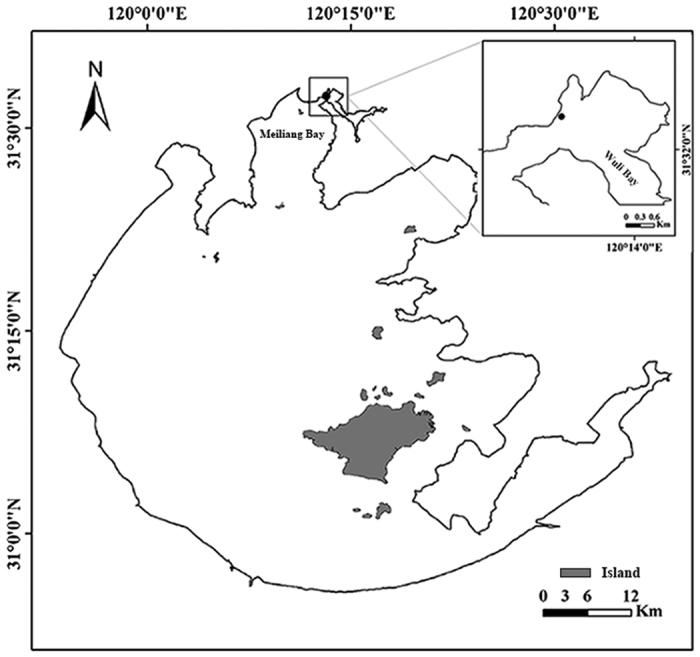
Map of Lake Taihu and study site (dot). This map was generated in ESRI ArcMap 10.2 (Environmental Systems Resource Institute, ArcMap 10.2 ESRI, Redlands, California, USA, http://www.esri.com/).

The sampling site (31°32′22.92″N, 120°13′9.98″E, Fig. 3) is located just before the sluice of Wuli Bay, near Wuxi City. Meiliang Bay of Lake Taihu is directly south of the study site and is hydrologically connected to the main body of the lake (Fig. 3). In a previous study, Cai *et al.*^30^ found that *T. chinensis* is the dominant benthic invertebrate species in this region, with a maximum abundance of ca. 1280 ind./m^2^. Due to strong monsoons, dense algae usually accumulate in this region during the bloom season^30^. The water in this area is severely polluted by industrial wastewater and domestic sewage from Wuxi City^49^, threatening human health due to consumption of aquatic resources by the local population.

### Sample collection

*T. chinensis* samples were collected monthly with a 0.025 m^2^ modified Peterson grab sampler from July 2013 to June 2014. Approximately 0.02 m^3^ of sediment (<5 cm depth) was collected and sieved *in situ* through a 250 μm aperture mesh size sieve. In the laboratory, *T. chinensis* specimens were carefully sorted on a white tray. Because cyanobacteria ingested by *T. chinensis* may simply pass through the gut without being assimilated, and thereby confound our results, we placed individuals in beakers with tap water for 24 hours to allow clearance of gut contents. Following clearance, all the specimens were stored in a 10 ml centrifuge tube at −80 °C until MC analysis.

Water and sediment samples also were collected monthly. Water samples were collected from the near surface (<0.5 m) with a 2.5 L plexiglass water sampler. A 0.5 L water sample, which was preserved with 1% acidic Lugol’s solution, was used for phytoplankton identification. Another 1 L water sample was used for intraMCs and extraMCs analysis (see below). A separate surface sediment sample also was taken using a Peterson grab sampler, and stored at −80 °C for analysis of MCs and other sediment-related parameters (see below). In addition, water samples of one additional month on July 2014 for phytoplankton identification and three additional months from July to September 2014 for extraMCs analysis were added, as replacements for the missing samples in 2013.

### Environmental parameters analysis

Temp, pH and Cond were measured in the field with a Yellow Springs instruments (YSI) 6600 V2 multi–sensor sonde (USA); SD was measured *in situ* with a Secchi disc (diameter 0.3 m). TN, NH_4_^+^–N, NO_2_^−^–N, NO_3_^−^–N, TP, PO_4_^3−^–P, DO, DTIC, DTOC, TSS and Chl–a data in our study area were obtained from a single station of the Taihu Laboratory for Lake Ecosystem Research (TLLER) within the Chinese Ecosystem Research Network (CERN), which has a regular monitoring program.

Samples used for DTIC, DTOC, Chl-a and TSS detection were filtered through Whatman GF/F filters. DTIC and DTOC were determined by the high temperature combustion method^50^. Briefly, filtered samples were separately injected into a high temperature combustion chamber and a low temperature reaction chamber; the generated CO_2_ is analyzed via a non-dispersive infrared absorption TOC analyzer. Finally, DTOC is obtained from the subtraction of DTIC from dissolved total carbon. Chl-a and TSS were analyzed according to the method described in ref. 51. Briefly, after extraction with 90% ethanol at 80 °C, chl-a was measured using a Shimadzu spectrophotometer at 665 nm and 750 nm with a correction for phaeopigments (Pa). Pre-combusted and pre-weighed Whatman GF/F filters were used to collect TSS of nominal sizes larger than 0.7 μm. Then, filters were dried and weighed for the calculation of the TSS concentrations.

TN, NH_4_^+^–N, NO_2_^−^–N, NO_3_^−^–N, TP, PO_4_^3−^–P and DO were determined according to standard methods^50^. TN was analyzed spectrophotometrically at 210 nm after digestion. According to the molybdenum blue method, TP was analyzed spectrophotometrically at 700 nm after digestion with alkaline potassium persulfate. NH_4_^+^–N, NO_2_^−^–N, NO_3_^−^–N and PO_4_^3−^–P concentrations were determined by flow injection analyzer (Skalar SAN++, The Netherlands). The iodometric method was used in DO determination. In detail, DO in samples were rapidly oxidized an equivalent amount of the dispersed divalent manganous hydroxide precipitate to hydroxides of higher valency states. In the presence of iodide ions in an acidic solution, the oxidized manganese reverts to the divalent state, with the liberation of iodine equivalent to the original DO content.

TN, TP, TOC and heavy metal (Cr, Cu, Fe, Mn, Ni and Zn) content in sediment were analyzed. After being freeze–dried and ground, heavy metal content in sediment was analyzed by ICP–AES (Teledyne Leeman LABS, USA), TOC was measured according to the method of dichromate oxidation^52^ and TN and TP were determined via potassium supersulphate oxidation–ultraviolet spectrometer^30^.

Phytoplankton identification and biomass calculation were based on the method of Niu *et al.*^53^.

### MCs analysis

MC contents in chironomid (*T. chinensis*) per dry weight (DW) were determined according to the method of Xie and Park^54^. Lyophilized *T. chinensis* samples were ground and extracted with 10 ml of BuOH: MeOH: H_2_O (1:4:15), sonicated for 3 min, then centrifuged at 10000 r/min (15 min at 4 °C). Extraction, sonication and centrifugation were repeated three times, and the supernatant was then added to a pre–activated HLB cartridge (200 mg, Oasis^®^, Waters, Milford, MA, USA). When the liquid level in the cartridge was nearly gone, 20 ml of 5% MeOH and 12 ml of 100% MeOH were added to wash and elute, respectively. The eluent was then dried under N_2_ at 40 °C, and the residue was re–dissolved in 100% MeOH; this solution was added to a preconditioned silica gel cartridge (2 g)/plus silica gel (0.69 g) tandem cartridge (Waters, Milford, MA, USA). Finally, after washing and eluting with 100% and 70% MeOH, respectively, the remaining residue after drying was re–dissolved in 300 μl MeOH for High–Performance Liquid Chromatography (HPLC) analysis.

MC concentrations in sediment, which also may include MCs from benthic cyanobacteria, were analyzed following the method of Chen *et al.*^55^ using an extraction solvent of 0.1 M EDTA and 0.1 M Na_4_P_2_O_7_ (acidified with 0.1% TFA, v/v). Water column samples were used for both intraMCs and extraMCs analysis. They were first filtered through a glass microfiber filter (GF/C, Whatman, UK). The filter paper and filtrate were used for the determination of intraMCs and extraMCs concentrations, respectively. Filter papers were lyophilized, and then sonicated in 5% acetic acid. After centrifugation of the samples, intraMCs and extraMCs (filtrate was directly applied to HLB cartridge) analyses followed the method of Su *et al.*^56^.

All concentrated samples were analyzed by HPLC (Agilent 1200 series, Palo Alto, CA, USA) to determine the concentrations of three MC congeners (MC–LR, MC–RR and MC–YR)^56^. Total MC concentrations (TMCs) represent the sum of the three MC congeners. Standards for the three MC congeners were obtained from Sigma-Aldrich (München, Germany).

### Data analysis

All the statistical analyses were conducted using IBM SPSS version 20 (USA). Average, maximum and minimum values of various environmental parameters for 12 months were performed. Spearman’s correlation tests were conducted to examine the multiple correlations between various environmental parameters and CMCs. In order to avoid high collinearity among variables, correlation coefficients that exceeded 0.8 in Spearman’s correlations were excluded. This resulted in the removal of 16 variables from the final stepwise multiple linear regression analysis (MLR). The stepwise MLR was used to identify the key parameters associated with the temporal variation of MC accumulation in *T. chinensis*. The statistical significance levels were set as 0.05 and 0.10 for variable inclusion and removal. Only the data that were obtained during the months of July 2013 to June 2014 were used in the statistical analysis. All the variables were standardized by SPSS before data analysis. In addition, TSI values were calculated with the method of Cai *et al.*^57^ and Carlson^58^, based on the parameters of chl-a, SD and TP.

## Conclusion

Despite numerous studies that have focused on MC accumulation in aquatic organisms, this study is the first report on MC bioaccumulation in *T. chinensis*. Our study indicates that *T. chinensis* could accumulate high concentrations of MC during hot summer months, except during times when *T. chinensis* are emerging. MC concentrations in *T. chinensis* were several orders of magnitude higher than that in bivalvia, gastropods, shrimp and fish from the same lake. During the survey, MC-RR was the most abundant congener in *T. chinensis*, whereas MC-LR accounted for most of the intraMC and dominated the other two MC sources, suggesting that among the three congeners, MC-LR may be depurated the most efficiently by *T. chinensis*. In addition, intraMC was the main source for MC accumulation in *T. chinensis*. Meanwhile, cyanobacteria biomass explained the most variation with respect to total MCs accumulated in *T. chinensis*. Because of the high concentrations of MC in *T. chinensis* and the possibility of food web transfer, routine monitoring is recommended to avoid potential health threats.

## Additional Information

**How to cite this article**: Xue, Q. *et al.* Accumulation of microcystins in a dominant Chironomid Larvae (*Tanypus chinensis*) of a large, shallow and eutrophic Chinese lake, Lake Taihu. *Sci. Rep.* **6**, 31097; doi: 10.1038/srep31097 (2016).

## Figures and Tables

**Table 1:** Temporal dynamics of environmental parameters in the study site from July 2013 to June 2014.

	**Parameters**	**Mean**	**Minimum**	**Maximum**
Water column	Temp (°C)	19.22	4.61 (2)	30.93 (7)
	pH	8.04	7.42 (11)	8.69 (7)
	DO (mg/L)	8.84	5.43 (8)	11.25 (3)
	Cond (μs/cm)	525	385 (1)	745 (6)
	SD (m)	0.4	0.14 (10)	0.8 (3)
	Chl-a (μg/L)	42.42	7.23 (2)	115.53 (10)
	PO_4_^3−^-P (mg/L)	0.017	0.001 (4)	0.071 (8)
	TP (mg/L)	0.14	0.05 (12)	0.28 (7)
	NO_2_^−^-N (mg/L)	0.022	0.003 (9)	0.094 (7)
	NO_3_^−^-N (mg/L)	0.37	0.07 (10)	0.64 (3)
	NH_4_^+^-N (mg/L)	0.39	0.24 (4)	0.53 (11)
	TN (mg/L)	2.25	1.18 (12)	3.69 (6)
	DTIC (mg/L)	21.54	17.88 (7)	29.19 (5)
	DTOC (mg/L)	4.63	2.18 (3)	6.45 (8)
	TSS (mg/L)	33.51	12.2 (4)	65.87 (8)
	TSI	67.83	58.6 (2)	81.5 (10)
Sediment	TN (g/kg)	2.56	1.96 (7)	2.93 (11)
	TP (g/kg)	0.61	0.47 (7)	0.69 (6)
	TOC (%)	1.9	1.65 (1)	2.19 (11)
Phytoplankton	Cyanobacteria (mg/L)	2.69	0 (2)	15.26 (7)
	*Crytophyta* (mg/L)	0.35	0 (6)	2.21 (11)
	*Bacillariophyta* (mg/L)	0.4	0.006 (11)	1.12 (1)
	*Euglenophyta* (mg/L)	0.04	0 (9,11,4)	0.24 (3)
	*Chlorophyta* (mg/L)	1.97	0 (12)	16.93 (9)

Numbers in parentheses: the months (1 = January, etc.) that the minimum and maximum values were measured. Temp: water temperature; DO: dissolved oxygen; Cond: conductivity; SD: Secchi depth; Chl-a: chlorophyll a; PO_4_^3−^-P: orthophosphate; TP: total phosphorus; NO_2_^−^-N: nitrite; NO_3_^−^-N: nitrate; NH_4_^+^-N: ammonium; TN: total nitrogen; DTIC: dissolved total inorganic carbon; (D)TOC: (dissolved) total organic carbon; TSS: total suspended solid particles; TSI: trophic status index.

**Table 2:** Correlations between CMCs concentrations and different environmental parameters (only ones that are statistically significant or R ≈ 0.5 are included).

Parameters	CMC-LR	CMC-RR	CMC-YR	CTMC
IntraMC-LR	0.730**	0.662*	0.730**	0.662*
IntraMC-RR	0.710**	0.832**	0.710**	0.815**
IntraMC-YR	0.694*	0.549	0.694*	0.577*
Intra-TMC	0.701*	0.669*	0.701*	0.662*
ExtraMC-RR	−0.821**	−0.667*	−0.821**	−0.776**
Temp	0.552	0.606*	0.552	0.606*
pH	0.634*	0.599*	0.634*	0.599*
DO	−0.473	−0.528	−0.473	−0.500
TP	0.545	0.528	0.545	0.577*
DTIC	−0.598*	−0.479	−0.598*	−0.493
SD	−0.522	−0.721**	−0.522	−0.689*
Chl-a	0.559	0.683*	0.559	0.676*
TSI	0.626*	0.732**	0.626*	0.746**
Fe	0.497	0.472	0.497	0.472
*Microcystis*	0.634*	0.782**	0.634*	0.745**
Cyanobacteria	0.572	0.706*	0.572	0.688*
*Bacillariophyta*	−0.572	−0.706*	−0.572	−0.716*
*Euglenophyta*	−0.546	−0.326	−0.546	−0.417

^*^Correlation is significant at the 0.05 level (2-tailed);

^**^Correlation is significant at the 0.01 level (2-tailed). IntraMC (-LR, -RR, -YR): intracellular MC (-LR, -RR, -YR); intra-TMC: total intracellular MC. ExtraMC (-LR, -RR, -YR): extracellular MC (-LR, -RR, -YR). CMC (-LR, -RR, -YR): MC (-LR, -RR, -YR) in *T. chinensis*; CTMC: total MC in *T. chinensis*.

**Table 3:** The results of stepwise multiple regression analysis for MCs in *T. chinensis* (dependent variables) and environmental parameters (independent variables).

	CMC-LR	CMC-RR	CMC-YR	CTMC
	Std. Coefficient	Std. Coefficient	Std. Coefficient	Std. Coefficient
Cyanobacteria	0.807	—	—	0.889
*Oscillatoria*	—	−0.344	—	—
IntraMC-RR	—	1.294	—	—
ExtraMC-LR	0.319	—	—	0.197
ExtraMC-YR	—	—	0.324	—
TN	—	—	1.284	—
NO_3_^−^-N	—	—	−0.466	—
NO_2_^−^-N	0.061	—	—	0.054
Adjusted R^2^	1	0.995	0.975	1
P	<0.01	<0.01	<0.01	<0.01
VIF	<3	<5	<3	<3

VIF: variation inflation factor.
